# Identification and quantification of spinochromes in body compartments of *Echinometra mathaei*’s coloured types

**DOI:** 10.1098/rsos.171213

**Published:** 2018-08-22

**Authors:** Lola Brasseur, Marie Demeyer, Corentin Decroo, Guillaume Caulier, Patrick Flammang, Pascal Gerbaux, Igor Eeckhaut

**Affiliations:** 1 Biology of Marine Organisms and Biomimetics Unit, Research Institute for Biosciences, University of Mons—UMONS, 23 Place du Parc, 7000 Mons, Belgium; 2 Organic Synthesis and Mass Spectrometry Laboratory, Research Institute for Biosciences, University of Mons—UMONS, 23 Place du Parc, 7000 Mons, Belgium

**Keywords:** spinochromes, pigments, polyhydroxy-1, 4-naphthoquinones, *Echinometra mathaei*, sea urchin, mass spectrometry

## Abstract

Sea urchin pigmentation is mainly due to polyhydroxy-1,4-naphthoquinones called spinochromes. If their molecular structures are well known in test and spines of many species, their abundance and distribution in other body compartments remain unstudied. The aim of this study is to analyse the pigment composition in four body compartments (test/spines, digestive system, gonads and coelomic fluid) of four coloured types of the sea urchin *Echinometra mathaei*. Qualitative and quantitative measurements by mass spectrometry highlight the existence of 13 different pigments; among which are five isomers of known spinochromes as well as three potentially new ones. The composition comparison shows the largest spinochrome diversity in ‘test/spines’ body compartments. The spinochrome concentrations vary from 48 to 1279 mg kg^−1^ of dried body compartment. It is the highest in the digestive system, although it is also important in the organic fraction of the ‘test/spines’ body compartment. This observation may be explained by higher exposures of some body compartments to external environments and by the protective role fulfilled by spinochromes against microorganisms, ultraviolet radiation and reactive oxygen species. The ‘black’ type—the most common coloured type in coral reefs—has the highest concentration of spinochromes indicating their importance in Echinoids' fitness by acting as a protective agent.

## 1. Introduction

The Echinoderm phylum constitutes an animal group that has a strong impact in marine environments. It includes well-known organisms such as sea cucumbers, sea stars, sea lilies, brittle stars and sea urchins. These animals are known to produce various secondary metabolites, whose specificity matches with their taxonomic classes [1,2]. For example, sea urchins produce naphthoquinones, also called spinochromes or echinochromes, that are involved in pigmentation of their tests, spines and internal organs. To date, more than 40 naphthoquinones have already been extracted and identified from sea urchins [3–7] since their discovery in the genus *Echinus* by MacMunn in 1883 [8]. They are generally classified by letters (i.e. Echinochrome A and Spinochromes A–E) for the most common ones and are generally considered as derivatives of polyhydroxy-1,4-naphthoquinones (PHNQ) substituted with some ethyl, acetyl, methoxy or amino groups [2,5,9]. Moreover, several spinochrome dimers were also discovered in sea urchins [10,11].

Though their diversity and molecular structures are well studied, their biological role remains largely hypothetical. However, as they are highly concentrated in external parts and given their potentially highly reactive molecular structure, the spinochromes are most probably involved in the protection of sea urchins. In this way, some studies suggest that PHNQ are involved in the defence mechanism against biofilm formation due to their antibacterial and fungicidal properties [6,12–16]. Some other studies further suggest a role as protective agents against UV-induced damage, due to their capacity to absorb UV light, and their antioxidant activity [16–19]. Finally, some studies showed the involvement of pigment cells in immune defence [20,21]. Moreover, recent findings about the use of antioxidants to scavenge the reactive oxygen species (ROS) implied in various diseases (e.g. cancer, allergy, degenerative diseases, diabetes, arteriosclerosis or ageing process), suggest that spinochromes, based on their high antioxidant potential, could provide food for thought in pharmaceutical industries [7,14,22,23]. Recently, one of these pigments, namely Echinochrome A, has been introduced as the active component in a drug called Histochrome and administered against ophthalmic diseases [3,4,7] and as prevention drug against myocardial infarction [24].

Our research focused on the sea urchin *Echinometra mathaei* (Blainville, 1825), also known as the burrowing urchin, a common echinoid from the Echinometridae family distributed throughout coral reefs and shallow waters in Indo-Pacific oceans [25]. Some studies already revealed some differences inside the *E. mathaei* species like the ecological distribution, adult morphology, larvae morphology, karyotype or gametes [26–32], and today it is commonly accepted that *E. mathaei* contains different types of population. Four morphotypes, named morphotypes A, B, C and D, are distinguished according to their morphology and their ecological distribution [32]. Several colours are also observed across the types, but the most common are black, purple, brown and green.

The present study is the first to give information on the pigment composition of body compartments from various *E. mathaei* coloured types. Considering the protective role of spinochromes and the high abundance of the black type in nature, we hypothesize that spinochromes are more abundant and/or diversified in this type than in the others. The aim of this study is to investigate the spinochrome composition of the *E. mathaei*'s coloured types in four body compartments (test/spines, digestive system, gonads and coelomic fluid). The results will deepen the knowledge about the molecular diversity of PHNQ and will highlight the potential role of PHNQ in sea urchin fitness by linking the PHNQ composition to sea urchin type abundance in nature.

## 2. Material and methods

### 2.1. Samplings

**Figure 1.**
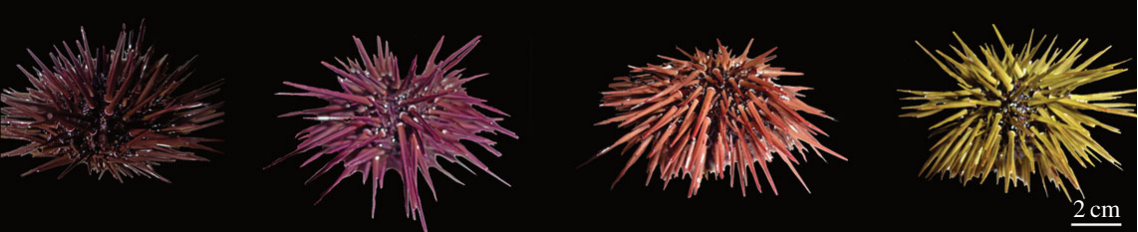
Coloured types of the collected *E. mathaei*. From right to left: black, purple, brown and green.

*Echinometra mathaei* (Blainville, 1825) individuals were randomly collected by snorkelling in shallow water at high tide on the Great Coral Reef of Toliara, Madagascar (23°23'34″ S, 43°38'47″ E) in November 2015. The criteria described in the literature seem to indicate that this population corresponds to type C of *E. mathaei* (colour variable, spine tip not white, white and clear basal ring of spine and intertidal distribution). In total, 345 individuals were observed and their coloured type recorded without distinction of sex, age or size and classified into one of the four coloured types: black, purple, brown and green (figure 1). A Pearson's *χ*^2^ test was performed to determine whether the general distribution of *E. mathaei's* coloured types is significantly different from a theoretically balanced distribution (i.e. 25% per type). Binomial tests were also carried out between each coloured type to determine which one is significantly different. Statistical analyses were performed using the ‘Prism 6’ software (GraphPad). Data visualizations were designed using ‘Microsoft Excel 2016’ (Microsoft Office) and Affinity Designer software.

Five individuals of large size (test diameter > 4 cm) from each coloured type were also collected. Once in the laboratory, they were weighed, measured and dissected. The spinochromes of the four body compartments of each individual were analysed: the digestive system, gonads, coelomic fluid and the ‘test/spines’ body compartment. The ‘test/spines’ body compartment consists of the mineral and organic part forming the body wall at the level of the test and the spines of the sea urchins. The digestive system, gonads and the ‘test/spines’ body compartment were washed in cold water and weighed after drying at 90°C during 24 h. Coelomic fluid was also collected by hole openings, then dried and weighed. The samples were stored in the dark at 5°C before spinochrome extraction.

### 2.2. Extraction of polyhydroxy-1,4-naphthoquinones

PHNQ of five sea urchins from each coloured type listed above were extracted, except for digestive system and gonads of ‘black’ type where only four individuals were extracted. Each dried body compartment (maximum 5 g of dried test/spines) was crushed and macerated in 10 ml of aqueous HCl 6M for 1 h before filtration under vacuum with a Buchner flask. The solution was partitioned three times against diethyl ether (v/v). The diethyl ether phases were pooled and partitioned three times against 30 ml of an aqueous NaCl 5% solution. Then, the ethereal phase was recovered and evaporated to dryness at low pressure at 60°C using a rotary evaporator (Laborota 4001 efficient, Heidolph), re-dissolved in 80% methanol and centrifuged at 10^4^*g* for 10 min. Finally, each sample was evaporated to dryness using a Speed Vac (RC 10.22, VWR international) and weighed.

### 2.3. Mass spectrometry analyses

PHNQ were analysed by LC-MS. For that purpose, a Waters Alliance 2695 liquid chromatography device (HPLC) was used. The system comprises a quaternary pump, a vacuum degasser and an autosampler. The chromatography was performed on reversed phase column (Kinetex^®^ C8 5 µm Biphenyl 100 Å, 50 × 4.6 mm, Phenomenex) at 30°C and with a sample volume injected of 10 µl and a constant flow (1.25 ml min^−1^) of a gradient of eluent A (water, 0.1% formic acid) and eluent B (acetonitrile) (table 1). The HPLC device was coupled to a different mass spectrometer according to the kind of analysis (see below for details). PHNQ could not be characterized by tandem mass spectrometry (MS/MS). The tandem mass spectrometry experiments are conducted under low kinetic energy condition, below 100 eV. These conditions are defined by the mass spectrometer used for the analysis (QToF MS). Therefore, the internal energy transferred to the mass selected ions remains too weak to generate dissociation reactions involving the backbone of the cyclic ions under investigation. Also, the substituents (OH groups) are so strongly appended on the cyclic carbon atoms to be expelled through single bond cleavage processes.

**Table 1.** Gradient timetable used for the HPLC analyses.

time (min)	eluant A (%)	eluant B (%)	curve
−10 → 0	80	20	equilibration
0 → 15	80 → 50	20 → 50	linear gradient
15 → 16	50 → 80	50 → 80	linear gradient
16 → 18	80	20	isocratic

#### 2.3.1. Accurate mass measurements

Accurate mass measurements and molecular formula of PHNQ ion predictions were performed on a Waters Q-ToF Premier using the electrospray ionization (ESI) source in the negative ionization mode, by scanning between *m/z* 50 and 600 with scan durations of 1 s and an inter-scan time of 0.1 s. The ESI conditions were as follows: capillary voltage of 3.1 kV, cone voltage of 40 V, source temperature at 120°C and desolvation temperature 300°C. Dry nitrogen was used as ESI gas with a flow rate of 50 l h^−1^ for the gas cone and 600 l h^−1^ for the desolvation gas. The mass spectrometer was equipped with a lockspray set-up to determine the exact masses of PHNQ ions. Sodium iodide was used as the reference sample, in particular *m/z* 126.9045 (I^−^) used as lock mass. Chemical structures were proposed from the available literature and correlated to the accurate mass measurements. Naphthoquinones structures were finally drawn using ‘ChemDraw 15.0.0.106’ (PerkinElmer Informatics, Inc.) software. The spectrum, the chromatogram and the chemical structures were annotated with Affinity Designer software.

#### 2.3.2. Quantification

For the quantitative part of the study, the internal standard (IS) method was selected and 2-hydroxy-1,4-naphthoquinone was privileged as the IS based on the similarity between its chemical structure and the PHNQ. The IS stock solution was prepared in 80% methanol at a concentration of 10 µg ml^−1^ and directly added to the dried sample. The mass spectra were obtained on a Waters Quattro Ultima using the ESI source in the negative mode by scanning between *m/z* 50 and 1500. The ESI conditions were as follows: capillary voltage of 3.1 kV, cone voltage of 40 V, source temperature at 120°C and desolvation temperature at 300°C. Dry nitrogen was used as the ESI gas with a flow rate of 50 l h^−1^ for the gas cone and 500 l h^−1^ for the desolvation gas.

#### 2.3.3. Mass spectra analyses and visualization

Mass spectra analyses were performed using MassLynx 4.1 mass spectrometry software (Waters). Relative PHNQ concentrations were measured with QuanLynx 4.1 mass spectrometry software (Waters). The intensities were normalized first to the IS intensity before being normalized with the dried body compartment weight to be compared. Statistical analyses were performed with the ‘Prism 6’ (GraphPad) software. Global PHNQ values were statistically compared with one-way ANOVA and Bonferroni's multiple comparisons tests.

## 3. Results

### 3.1. Distribution of coloured types

**Figure 2.**
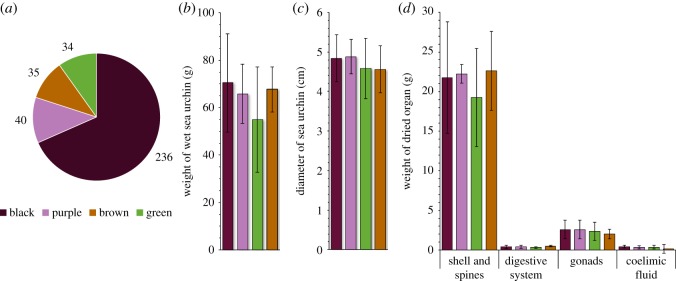
Description of (*a*) *E. mathaei* type population in Toliara bay (Madagascar), (*b*) wet weight (g) of sampled individuals of *E. mathaei* (*n* = 5), (*c*) diameter (cm) of sampled individuals of *E. mathaei* (*n* = 5), (*d*) dried body compartment weight (g) of sampled individuals of *E. mathaei* (*n* = 5).

Among the 345 individuals of *E. mathaei*, 236 were notified as ‘black’ (68.4%), 40 as ‘purple’ (11.6%), 35 as ‘brown’ (10.1%) and 34 as ‘green’ (9.9%) (figure 2). *χ*^2^ statistical analysis revealed that the distribution of types is significantly different from the theoretical distribution (*p* < 0.0001), the null hypothesis being that the different types are equally likely. Binomial tests performed between coloured types show that only the ‘black’ type is significantly different from others (*p* < 0.0001). The tests also show that no difference was found between ‘purple’, ‘green’ and ‘brown’ types (*p* > 0.05).

### 3.2. Distribution of polyhydroxy-1,4-naphthoquinones among *E. mathaei* body compartments and coloured types

Upon mass spectrometry analyses, 13 different PHNQ peaks, corresponding to the PHNQ ions listed in table 2, are detected from the different *E. mathaei* body compartment extracts.

**Table 2.** PHNQ molecules detected in different body compartment and coloured types of *E. mathaei* (new molecules are highlighted in italics). MW represents the theoretical mass, whereas [M–H]^−^ is the measured mass.

no.	PHNQ	retention time (min)	MW (U)	[M–H]^−^ *m/z*	predicted formula^a^	number of functional groups^b^					
						OH	CH_3_	NH_2_	COCH_3_	C_2_CH_5_	OCH_3_
1	Spinochrome B	2.75	222.02	221.01	C_10_H_6_O_6_	4					
2	Spinochrome D—Iso 1	2.65	238.15	237.00	C_10_H_6_O_7_	5					
3	Spinochrome D—Iso 2	6.68	238.15	237.04	C_10_H_6_O_7_	5					
4	Spinochrome D—Iso 3	8.65	238.15	237.04	C_10_H_6_O_7_	5					
*5*	*Spinochrome 252*	*3.72*	*252.18*	*251.02*	*C_11_H_8_O_7_*	*5*	*1*				
*6*	*Echinamine 253*	*2.41*	*253.16*	*252.16*	*C_10_H_7_O_7_N*	*5*		*1*			
7	Spinochrome E	1.73	254.15	252.99	C_10_H_6_O_8_	6					
8	Spinochrome A—Iso 1	6.09	264.19	263.01	C_12_H_8_O_7_	4			1		
9	Spinochrome A—Iso 2	7.95	264.19	263.01	C_12_H_8_O_7_	4			1		
10	Echinamine A/B	9.52	265.22	264.03	C_12_H_11_O_6_N	4		1		1	
11	Echinochrome A	6.69	266.04	265.03	C_12_H_10_O_7_	5				1	
12	Spinochrome C	6.73	280.18	279.01	C_12_H_8_O_8_	5			1		
*13*	*Spinochrome 282*	*6.72*	*282.20*	*281.03*	*C_12_H_10_O_8_*	*4*					*2*

^a^Based on accurate mass measurements.

^b^Functional groups on the PHNQ. The functional groups of potentially new PHNQ remain hypothetical.

**Figure 3.**
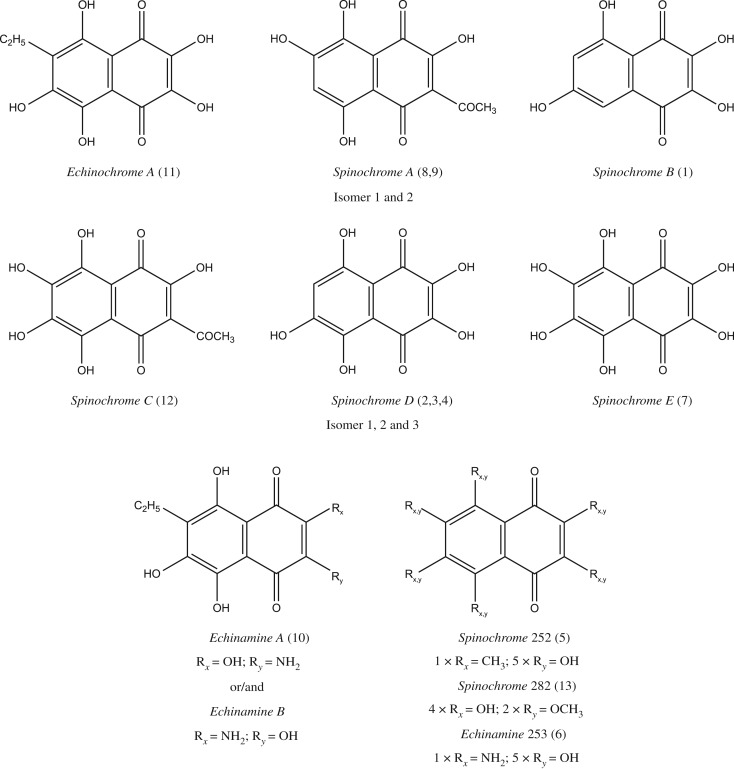
PHNQ molecular structures from *E. mathaei*. Isomers of the Spinochrome A and the Spinochrome D are not represented in this figure. The Echinamine A and B are both represented as we cannot make a clear-cut distinction. The potentially new PHNQ are represented with their hypothetical defined functional groups. The annotation is defined as the number of functional groups × R_position on figure_ = functional group.

For the identification of the different spinochrome molecules, the experimental masses could be correlated to the expected masses corresponding to spinochrome congeners and we proposed some molecular structures in table 2 based on the literature data [2,7,33]. Doing so, we observed that 10 molecules could match with already known PHNQ: Echinochrome A (11), Spinochrome A (8–9), Spinochrome B (1), Spinochrome C (12), Spinochrome D (2–4), Spinochrome E (7) and Echinamine A/B (10) (figure 3). The distinction between the isomeric Echinamine A/B is not achievable based only on the accurate mass measurements. However, it is interesting to note that only one LC signal is observed for this composition (at *m/z* 264) revealing that (i) only a single isomer is present or (ii) both isomers are perfectly coeluting.

**Figure 4.**
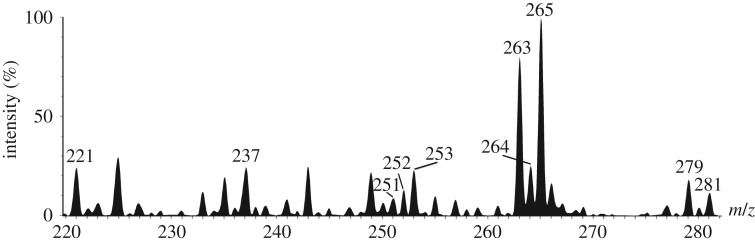
Full scan (−) mass spectrum of the PHNQ extract from test and spines from the ‘black’ type of *E. mathaei* (third replicate). MS signal annotated by ion mass corresponds to PHNQ molecules identified in *E. mathaei* and listed in table 2.

Among them, five potential isomers—same ion composition appearing at different retention times—of known pigments are found: three for Spinochrome D (2–4) and two for Spinochrome A (8–9). Moreover, three potentially new PHNQ are suspected to be present based on their predicted molecular formula (HRMS). Based on structure similarities, we arbitrarily named these new molecules as follows: Spinochrome 252 (5), Echinamine 253 (6) and Spinochrome 282 (13), with the number after the molecule generic name being the measured molecular mass. The Echinamine 253 (6) was recently shown in another paper as a new putative sea urchin echinamine but, as shown here, only identified with a predicted formula [16]. Figure 4 shows a representative example of a full scan mass spectrum (averaged on the retention time full window) obtained when analysing by LC-MS the pigment extract prepared from the spines of the ‘black’ type. The other non-identified ions (not labelled in the figure) did not show a defined retention peak on the chromatogram and/or did not have a predicted molecular formula matching with a PHNQ derivative.

**Figure 5.**
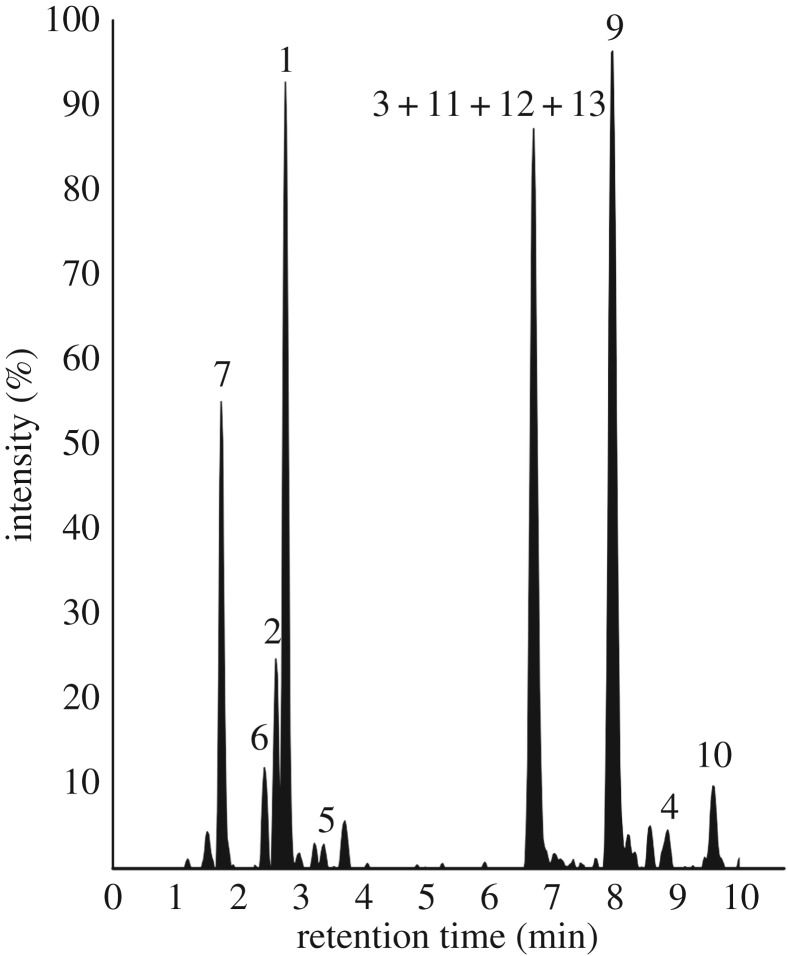
LC-MS analysis of the PHNQ extract from test and spines from the ‘black’ type of *E. mathaei* (third replicate). Peaks are annotated by numbers assigned to PHNQ molecules identified in *E. mathaei* and listed in table 3. The chromatogram time window is limited to the first 10 min in this figure for clarity.

Whereas the distribution of most of the PHNQ have a good resolution (figure 5), four pigments seem to coelute: the Spinochrome D—Iso 2 (3), the Echinochrome A (11), the Spinochrome C (12) and the Spinochrome 282 (13). The first pigment to be eluted is identified as the Spinochrome E (7), a molecule with six hydroxyl groups. The second one is a potentially new echinamine, the Echinamine 253 (6) with five hydroxyl groups and one amine group. The third is the Spinochrome D—Iso 1 (2) with five hydroxyl groups directly followed by the Spinochrome B (1) with four hydroxyl groups. Then, the Spinochrime 252 (5) can be found with potentially five hydroxyl and one methyl groups. Between 6 and 7 min, the coelution of four pigments can be detected with five hydroxyl groups for the (3), five hydroxyl and one ethyl groups for the (11), five hydroxyl and one acetyl groups for (12) and four hydroxyl and one methoxy groups. Spinochrome A—Iso 2 (9) presenting with four hydroxyl and one acetyl groups is then eluted after 8 min. Finally, the Spinochrome D—Iso 3 (4) with five hydroxyl groups can be detected followed by the Echinamine A/B (10) with four hydroxyl, one amine and one ethyl groups (10).

**Table 3.** Detailed concentrations of PHNQ contents in the body compartments from different coloured types of *E. mathaei* (mg of PHNQ by kg of dried body compartment). The intensities of the PHNQ ions observed in the mass spectrum, the IS intensity and the dried body compartment weight are used to calculate the main normalized content of each PHNQ molecules. The intensities are represented as mean ± standard deviation. Concentrations in italics correspond to the PHNQ molecules under the 1% threshold.

*N*^a^	black	purple	green	brown	black	purple	green	brown
	test with spines				digestive system			
1	9.17 ± 6.95	0.33 ± 0.23	6.19 ± 6.58	0.92 ± 0.64	*0.00 ± 0.00*	*0.58 ± 0.47*	*0.00 ± 0.00*	*1.77 ± 0.95*
2	2.14 ± 1.10	0.66 ± 0.24	1.17 ± 1.00	0.78 ± 0.31	43.42 ± 26.03	22.04 ± 9.38	35.16 ± 26.41	41.44 ± 27.80
3	0.71 ± 0.44	0.90 ± 0.28	0.68 ± 0.25	1.23 ± 0.32	37.69 ± 29.54	57.62 ± 52.82	46.15 ± 25.86	30.97 ± 9.52
4	*0.26 ± 0.31*	*0.00 ± 0.00*	*0.05 ± 0.05*	*0.06 ± 0.02*	807.12 ± 749.32	481.26 ± 240.87	707.65 ± 368.14	206.99 ± 130.47
5	*0.05 ± 0.03*	*0.06 ± 0.02*	*0.08 ± 0.03*	*0.13 ± 0.09*	*11.07 ± 5.60*	*8.66 ± 5.89*	*9.14 ± 6.44*	11.78 ± 6.73
6	1.40 ± 0.34	0.75 ± 0.34	0.84 ± 0.46	1.30 ± 0.29	*0.00 ± 0.00*	*0.00 ± 0.00*	*0.00 ± 0.00*	*0.00 ± 0.00*
7	4.85 ± 2.12	2.64 ± 0.86	2.53 ± 2.19	3.12 ± 2.18	*4.22 ± 5.72*	*6.64 ± 7.05*	*5.51 ± 3.08*	*7.54 ± 5.49*
8	0.52 ± 1.01	*0.10 ± 0.05*	*0.05 ± 0.06*	*0.06 ± 0.04*	*2.27 ± 1.89*	*2.02 ± 1.77*	*0.00 ± 0.00*	7.97 ± 7.92
9	13.02 ± 4.99	4.94 ± 2.93	2.79 ± 2.20	2.89 ± 2.24	*4.06 ± 3.31*	*3.16 ± 2.86*	*1.82 ± 1.21*	*2.16 ± 1.00*
10	0.50 ± 0.43	0.46 ± 0.29	0.64 ± 0.58	0.61 ± 0.46	*1.59 ± 1.78*	*4.61 ± 5.46*	*11.70 ± 9.59*	*3.38 ± 2.06*
11	10.32 ± 2.18	8.00 ± 1.90	9.89 ± 2.96	12.99 ± 2.08	303.41 ± 275.70	370.11 ± 180.34	398.00 ± 199.46	335.07 ± 79.77
12	2.43 ± 1.19	0.43 ± 0.21	0.53 ± 0.43	0.67 ± 0.21	*1.10 ± 1.67*	*1.13 ± 1.16*	*0.00 ± 0.00*	*1.28 ± 0.87*
13	0.55 ± 0.28	0.51 ± 0.10	0.49 ± 0.13	0.64 ± 0.17	20.65 ± 14.33	15.26 ± 14.58	23.24 ± 15.01	13.91 ± 6.78
*T*^b^	48.24 ± 12.18	21.67 ± 5.54	27.76 ± 13.58	25.07 ± 4.12	1265.47 ± 914.54	1006.07 ± 333.37	1279.06 ± 354.26	678.49 ± 103.85
	gonads				coelomic fluid			
1	*0.00 ± 0.00*	*0.09 ± 0.08*	*0.00 ± 0.00*	*0.00 ± 0.00*	*0.56 ± 0.62*	*0.00 ± 0.00*	*0.34 ± 0.52*	*0.00 ± 0.00*
2	1.58 ± 1.33	0.69 ± 0.74	1.30 ± 1.30	0.76 ± 0.52	13.73 ± 11.89	12.12 ± 17.67	5.16 ± 5.57	10.44 ± 12.25
3	5.22 ± 3.32	1.94 ± 1.57	4.18 ± 4.08	2.13 ± 1.64	27.92 ± 29.02	16.35 ± 15.23	10.06 ± 6.61	17.78 ± 17.98
4	48.33 ± 27.89	12.68 ± 7.04	7.47 ± 4.73	8.87 ± 5.32	8.29 ± 10.41	13.12 ± 5.43	13.80 ± 9.58	8.79 ± 4.05
5	*0.83 ± 0.97*	*0.30 ± 0.36*	*0.28 ± 0.26*	*0.58 ± 0.61*	*2.66 ± 3.23*	4.04 ± 3.44	*2.51 ± 2.39*	5.61 ± 4.89
6	*0.00 ± 0.00*	*0.00 ± 0.00*	*0.00 ± 0.00*	*0.00 ± 0.00*	*0.53 ± 0.45*	*0.00 ± 0.00*	*0.78 ± 1.74*	*0.75 ± 1.50*
7	1.49 ± 2.26	*0.38 ± 0.36*	1.93 ± 2.99	2.07 ± 2.59	9.54 ± 9.84	5.61 ± 6.45	3.03 ± 3.62	7.31 ± 10.15
8	*0.26 ± 0.15*	*0.12 ± 0.14*	*0.50 ± 0.43*	*0.19 ± 0.10*	8.59 ± 9.37	*1.73 ± 1.79*	*1.39 ± 1.71*	*2.67 ± 5.34*
9	*0.31 ± 0.25*	*0.12 ± 0.04*	*0.09 ± 0.11*	*0.14 ± 0.13*	*2.48 ± 2.05*	*1.01 ± 0.70*	*0.28 ± 0.27*	*1.24 ± 0.78*
10	*0.25 ± 0.27*	*0.11 ± 0.06*	*0.22 ± 0.36*	*0.00 ± 0.00*	*0.50 ± 0.33*	*0.67 ± 0.68*	*0.00 ± 0.00*	*2.56 ± 5.12*
11	60.90 ± 10.14	31.63 ± 13.35	52.63 ± 44.12	34.76 ± 16.26	219.50 ± 157.95	122.59 ± 49.38	152.18 ± 61.86	208.74 ± 129.31
12	*0.00 ± 0.00*	*0.00 ± 0.00*	*0.21 ± 0.23*	*0.12 ± 0.03*	*1.43 ± 1.64*	*2.30 ± 2.46*	*2.13 ± 1.89*	*1.18 ± 1.17*
13	1.77 ± 1.77	0.87 ± 0.64	2.02 ± 2.05	1.51 ± 0.86	7.26 ± 5.23	7.44 ± 5.77	4.46 ± 3.08	8.38 ± 6.08
*T*^b^	127.09 ± 23.41	54.14 ± 18.08	76.52 ± 54.09	57.68 ± 26.49	329.79 ± 194.42	214.23 ± 76.56	201.14 ± 42.10	310.39 ± 146.21

^a^Numbers assigned to PHNQ molecules identified in *E. mathaei* and listed in table 3.

^b^Total content of PHNQ molecules represented as mean of each replicate total content ± standard deviation.

**Figure 6.**
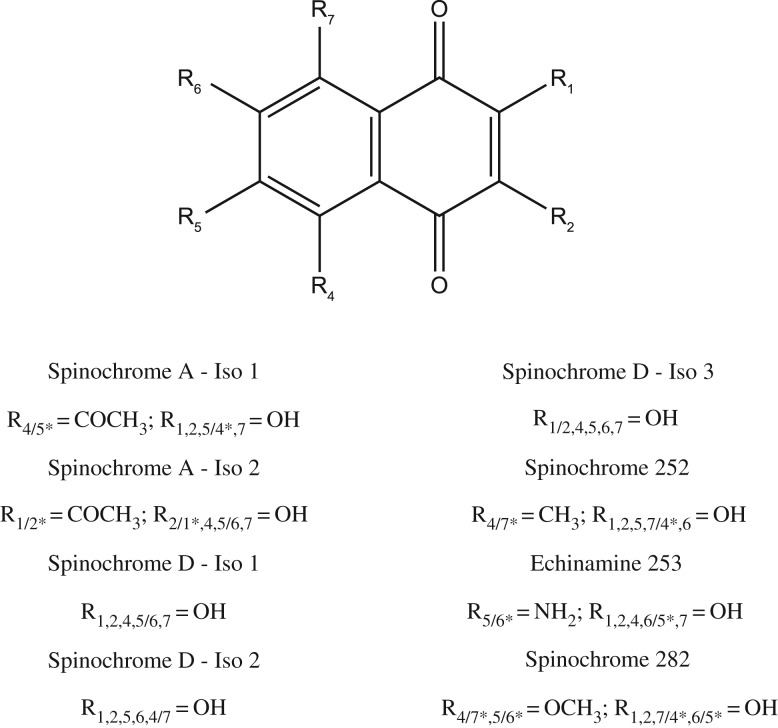
Hypothetical molecular structures of isomers and new PHNQ molecules from *E. mathaei* based on retention time. The annotation is defined as R_possible_ _position(s) on figure_ = functional group. The ‘/’ on R annotation shows the possibility of choice between two positions. The ‘,’ on R annotation shows all positions of the functional groups. The ‘*’ on R annotation means that if one position of the first functional group is chosen, the position of the other functional group has to be the complementary number (e.g. if the position of the methyl group on the Spinochrome 252 is the R_4_, the position of hydroxyl group will be the R_7_).

The retention time pattern (figure 5 and table 2) could bring some information about the molecular structures. Indeed, a first tendency can be observed: the retention time seems to decrease with the number of hydroxyl groups, which can be explained by their affinity with high water percentage in the elution solution at the beginning of the chromatography. A second tendency would be that the methyl, ethyl, acetyl or methoxy groups increase the retention time, which can also be explained by the decrease in their affinity with water. As for it, the amine group does not seem to have an influence on the retention time. However, some PHNQ do not follow these empirical rules and the different retention times for isomers suggest that the molecular conformation has an influence, too. Our hypothesis is that the presence of the functional group close to the oxygen influences the conformation, and so, the retention time. In this way, an amine group in R_1_ or R_2_ position would increase a lot the retention time (e.g. Echinamine A/B at 9.5 min). Indeed, even if this PHNQ owns an ethyl group which should slow its elution, the difference with the Echinamine 253 (6) (RT: 2.4) is too important. So, we suggest that the latter PHNQ has its amine group in position R_5_ or R_6_ (figure 6). In this way, we also suggest that an acetyl group in R_1_ or R_2_ would increase the retention time. Indeed, the analyses showed two isomers for Spinochrome A (at 6.1 min for Iso 1 (8) and at 8 min for Iso 2 (9)), and if we compare the corresponding retention times with Spinochrome C (12) (at 6.7 min), we can suppose that Spinochrome A (8–9) (with four hydroxyl groups), which has a similar conformation to Spinochrome C (12) (which has five hydroxyl groups), must be eluted after. So, we suggest that Spinochrome A—Iso 1 (8) has its acetyl group in R_5_ or R_6_ while Spinochrome A—Iso 2 (9) has it in R_1_ or R_2_. Afterwards, we suggest that a hydroxyl group in R_1_ or R_2_ would decrease the retention time. Indeed, Spinochrome D (2–4) has three isomers (at 2.6 min for Iso 1, at 6.7 min for Iso 2 and 8.6 min for Iso 3). As these PHNQ only have five hydroxyl groups, the difference of retention times between isomers must be linked to the position of the hydroxyl group. We hypothesize, as above, that the lack of hydroxyl group in R_1_ or R_2_ (replaced or not by another group) increases the retention time. So, we suggest that the first eluted Spinochrome D, Spinochrome D—Iso 1 (2) has its missing hydroxyl in R_5_ or R_6_, the last eluted, Spinochrome D—Iso 3 (4) has its missing hydroxyl group in R_1_ or R_2_. Concerning the second eluted, the Spinochrome D—Iso 2 (3), we suggest that it has its missing hydroxyl group in R_4_ or R_7_ where it can be still relatively close to oxygen to slow down the elution. With these hypotheses, we can also infer the potential molecular structure of the Spinochrome 252 (5) and Spinochrome 282 (13). Spinochrome 252 (5), with potentially one methyl and five hydroxyl groups, would have its methyl group in R_5_ or R_6_ because of its short elution time. The Spinochrome 282 (13), with potentially two methoxyl groups and four hydroxyl groups, would have its methoxyl groups in R_4_ or R_5_ and R_6_ or R_7_ due to its relatively short elution times. Needless to say, some NMR analyses would be necessary to confirm these hypotheses.

**Figure 7.**
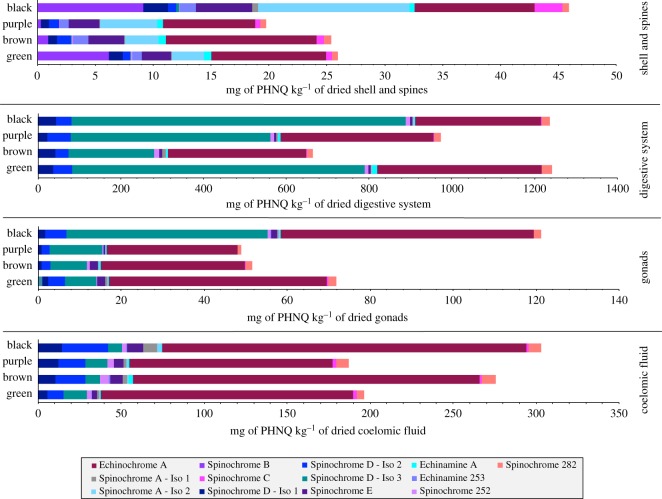
Comparison of the PHNQ contents of the body compartments from different coloured types of *E. mathaei*. The intensities of the PHNQ ions observed in the mass spectrum, the IS intensity and the dried body compartment weight are used to calculate the main normalized content of each PHNQ molecule. The exact values of means and their standard deviations are detailed in table 3.

To avoid a potential inter-body compartment contamination and to gain clarity, only pigments with a relative concentration threshold above 1% of total PHNQ content will be discussed below. The details of PHNQ main abundances and their standard deviation for each body compartment and type are listed in table 3 and illustrated in figure 7.

All the spine extracts present relatively similar compositions regardless of the types. They all mainly contain 10 PHNQ, say (1), (2), (3), (6), (7), (9), (10), (11), (12) and (13) as illustrated on the mass spectrum in figure 4. Among them, three are clearly dominant in all types: Echinochrome A (11) (around 20–50% of total content), Spinochrome A—Iso 2 (9) (around 10–25%) and Spinochrome E (7) (around 10%). Spinochrome B (1) is also highly concentrated but only in ‘black’ and ‘green’ animals. Global PHNQ concentrations are significantly similar in ‘purple’, ‘brown’ and ‘green’, around 20–25 mg of PHNQ per kilogram of dried spine extracts (*p* > 0.05), while the ‘black’ extracts appear significantly different (*p* < 0.05) and are almost two times more concentrated. This difference is especially due to Spinochrome A—Iso 2 (9) that is three times more concentrated than in other coloured types and also to Spinochrome B (1) that is between 10 and 30 times more concentrated than in ‘purple’ and ‘brown’ animal extracts.

The digestive system extracts also present similar compositions whatever the type but with less important diversity than the spine extract. They all mainly contain five PHNQ, say (2), (3), (4), (11) and (13). Moreover, ‘brown’ animal digestive system extracts also contain small quantities of Spinochrome 252 (5) and Spinochrome A—Iso 1 (8). Among them, two are clearly dominant in each coloured type: Spinochrome D—Iso 3 (4) (around 30–60% of total content) and Echinochrome A (11) (around 25–50%). Global PHNQ concentration is significantly similar for all types (*p* > 0.05). However, we observed a slightly higher concentration in ‘green’ and ‘black’ animals (around 1200 mg of PHNQ per kilogram of dried digestive system). ‘Purple’ animal extracts present a slightly lower concentration (around 1000 mg kg^−1^), but the concentration in ‘brown’ is clearly lower than in others (around 700 mg kg^−1^). The difference is mostly due to the variability of Spinochrome D—Iso 3 (4), the other pigment concentrations being relatively similar between the four coloured types.

The gonad extracts show again a similar composition whatever the types and with a relatively low diversity. They all contain five PHNQ: (2), (3), (4), (11) and (13). Moreover, all types except the ‘purple’ contain a small quantity of the Spinochrome E (7). Among them, two are clearly dominant in all types: the Spinochrome D—Iso 3 (4) (around 10–35% of total content) and the Echinochrome A (11) (around 35–65%). Although the concentration intra- and inter-body compartment is different, this distribution is relatively close to the digestive system. Indeed, the same PHNQ are found in both body compartments with a ratio of 10 between two total contents and a larger proportion for the Spinochrome D—Iso 3 (4) in the digestive system. Global PHNQ concentration is significantly similar in ‘purple’, ‘brown’ and ‘green’ (*p* > 0.05), around 50–70 mg of PHNQ per kilogram of dried gonads while the ‘black’ is significantly different (*p* < 0.05) except with ‘green’ (*p* > 0.05) and almost twice as concentrated. The difference is mostly due to the Spinochrome A—Iso 1 (8) which is five times more concentrated than in other types and due to the Echinochrome A (11) which is twice as concentrated as in ‘purple’ and ‘brown’.

The coelomic fluid extracts also show a relatively similar composition whatever the types. They all contain six PHNQ: (2), (3), (4), (7), (11) and (13). Moreover, ‘purple’ and ‘brown’ also contain small quantities of Spinochrome 252 (5) and the ‘black’ type is the only one that contains Spinochrome A—Iso 1 (8). Among them, only Echinochrome A (11) is clearly dominant in all types (around 55–75% of the total content). Global PHNQ concentrations are significantly similar (*p* > 0.05) in all types. However, the concentration seems slightly lower in ‘purple’ and ‘green’, around 190 mg of PHNQ per kilogram of dried coelomic fluid, while ‘black’ and ‘brown’ are closer and around 1.5 times more concentrated than other coloured types.

## 4. Discussion

The Toliara coral reef hosts a large variety of sea urchins and the species *E. mathaei* is especially abundant among them [34,35]. Various coloured types are found in the same location, but the present study shows that the ‘black’ one is largely predominant. It is likely that the difference between type abundances is explained by the survival rate and/or by the reproductive success of individuals, though we do not find any difference in the dried weight of the gonads between types, suggesting that the survival rate may be the most important factor between the two parameters. The major difference between coloured types is clearly the PHNQ concentration in body compartments, especially in test/spines and in gonads where the pigments are two times more concentrated in the ‘black’ type than in others. In the digestive system as well as the coelomic fluid, if the ‘black’ type always showed the highest concentration, the difference with other types is less clear. In this way, if we correlate this difference with the higher abundance of the ‘black’ type on Toliara coral reef, these observations may indicate that the PHNQ abundance in sea urchins has an impact on their fitness. In this way, a lot of studies show the high bioactive properties of PHNQ as antioxidant, antibacterial, antifungal, anti-inflammatory, UV-protector and ROS protector [7,14,18,21–24].

In the literature, PHNQ were only studied in the test, spines and the coelomic fluid [25,36,37], but, to our knowledge, other body compartments had never been analysed. Nevertheless, the coloration of the digestive system, gonads and coelomic fluid easily suggests the presence of PHNQ. At all, three potential new spinochromes were identified: the Echinamine 253, the Spinochrome 252 and the Spinochrome 282. The Echinamine 253 was already shown by Powell *et al*. [16] in 2014 as aminopentahydroxynaphthoquinone, but the structure was only based on predicted formula and not confirmed in NMR, as in this study. Then, our identification could confirm the presence of a potential new spinochromes already suggested by Powell and further studies with NMR would be needed to confirm the predicted structure.

The first major difference between body compartments (regardless of coloured types) is the larger PHNQ variety in test/spines than in other body compartments. This multiplication of different bioactive molecules suggests the need for high PHNQ diversity and again suggests a strong ecological role in the fitness of sea urchins [38]. The second major difference between body compartments is the huge concentration of PHNQ in the digestive system, as confirmed by microscopy in the literature [39]. However, the quantity of PHNQ being relative to dried body compartment, the test/spines concentration is misrepresented due to the importance of the mineral fraction of the sea urchin skeleton. Clarke & Wheeler [40] showed that sea urchin species morphologically close to *E. mathaei* contain 4–8% of organic matter in their test and spines. If the mineral fraction is taken into consideration, that would increase the PHNQ concentration to 600–1100 mg kg^−1^ of dried organic matter for the ‘black’ type and to 300–500 mg kg^−1^ for other types. A second correction should be done for coelomic fluid. The concentrations are relative to the dried coelomic fluids. Nevertheless, the coelomic fluid is mainly made up of water (around 30 ml (L Brasseur 2015, personal observations [35])) which induces the dilution of the PHNQ. Moreover, some studies have already shown that red spherule cells of coelomic fluid which contains spinochromes presented similar chemical properties to vertebrate mast cells of the immune system [41] and are recruited with the presence of bacteria [42–44]. In this way, we can estimate that the test/spines and the digestive system are clearly more concentrated in PHNQ than other body compartments which can be linked to their higher relation to the external environment and then seems to support the hypothesis of the function of PHNQ as a protective system.

The high concentration of PHNQ in the digestive system could also be explained by a possible involvement in the protection against microorganisms ingested with food. Furthermore, the PHNQ pattern of digestive system shows the predominance of Spinochrome D—Iso 3 while being less (or not at all) abundant in other body compartments, suggesting a potential role for this specific pigment. Finally, the presence of spinochromes in gonads suggests their implication in reproduction. Indeed, the presence of pigments was shown in the cortical part of sea urchin eggs of many species (e.g. *Arbacia punctulata*, *Lytechinus variegatus*, *Hemicentrotus pulcherrimus* or *Anthocidaris crassispina*) and their re-arrangement was observed after the fertilization with the migration of a part of spinochromes from the cortex to the centre of the embryo and a release of a part of pigments out of it [45–47].

## 5. Conclusion

This study is the first to highlight such a large diversity of pigments in *E. mathaei* in tests and spines and is the first to show the pigments diversity in other body compartments. The difference between PHNQ distribution and abundance in the four investigated body compartments suggests that PHNQ have a great importance in sea urchin biology and the variation of PHNQ between colour types may have an influence on their fitness and so, on populations. Further studies are needed to clearly determine the specific role, but the literature and the PHNQ composition in body compartments suggest a protective role against microorganisms and maybe also against UV radiation and ROS.

## Data accessibility

All data are available online [48].
